# Clinical Utility of Anorectal Manometry in Children with Functional Constipation: Can Anorectal Manometry Help Predict the Therapeutic Response?

**DOI:** 10.3390/children12040512

**Published:** 2025-04-16

**Authors:** Dhiren Patel, Courtney Decker, Leonel Rodriguez

**Affiliations:** 1 Division of Pediatric Gastroenterology, Hepatology and Nutrition, SSM Cardinal Glennon Children’s Medical Center, Saint Louis University School of Medicine, St Louis, MO 63104, USA; 2 Department of Pediatrics, Saint Louis University School of Medicine, St Louis, MO 63104, USA; deckerc@uiowa.edu; 3 Division of Pediatric Gastroenterology, Hepatology and Nutrition, New Haven Children’s Hospital, Yale University, New Haven, CT 06510, USA; leonel.rodriguez@yale.edu

**Keywords:** pediatrics, children, anorectal manometry, constipation, functional constipation, DGBI, functional GI disorders

## Abstract

**Background**: Anorectal Manometry (ARM) plays a crucial role in diagnosing potential motility disorders of anorectum in pediatric gastroenterology. Despite its prevalence, the predictive utility of ARM in guiding therapeutic response remains poorly characterized. **Objectives**: This study aims to evaluate the effectiveness of ARM in predicting therapeutic responses among children with functional constipation. **Methods**: A retrospective chart review was conducted at two tertiary centers examining pediatric patients who underwent ARM between January 2018 and July 2022. Key ARM parameters were analyzed, including anal resting pressure, recto-anal inhibitory reflex (RAIR), first rectal sensation, and bear-down maneuver (BDM). Therapeutic responses were assessed post-ARM, with success defined as an increase in bowel movement frequency and/or a decrease in fecal incontinence. In addition, we also intended to evaluate the eventual need for surgical intervention as another outcome. **Results**: The study included 327 patients, with a median age of 8.2 years. The overall therapeutic response rate was 40.7%, with stimulant laxatives showing a 48% response. Notably, lower anal resting pressures and delayed rectal sensations were associated with better therapeutic outcomes. Abnormal BDM correlated with a lack of response to therapies, while the presence of abnormal RAIR was linked to a higher eventual need for surgical intervention. **Conclusions**: ARM is instrumental in predicting therapeutic responses in pediatric patients with functional constipation. In addition to diagnosing HD, ARM could be an instrumental tool in identifying patients with dyssynergic defecation for early intervention with targeted therapy in age-appropriate patients.

## 1. Introduction

Gastrointestinal motility testing has become an indispensable tool in pediatric gastroenterology, aiding in the diagnosis and management of various motility disorders across the gastrointestinal (GI) tract. The evolution of motility tests has enabled comprehensive evaluation of GI contractility, enhancing our understanding of gastrointestinal function. Among these tests, Anorectal Manometry (ARM) stands out as the most frequently performed motility study in children, with functional constipation refractory to medical therapy being its predominant indication. ARM involves recording pressures during different maneuvers, including baseline assessments (resting pressure), challenge maneuvers (recto-anal inhibitory reflex, cough reflex, bear-down maneuver), and rectal sensation, offering insights into anal sphincter tone, anal rectal sensory response, anorectal reflexes, and rectal compliance [1]. While the literature and consensus papers delineate indications for ARM in pediatric patients, its role in predicting or guiding therapeutic response remains inadequately characterized in children. The paucity of research in this area could be due to the lack of prospective controlled studies, use of different equipment at various motility centers, non-standardized and unvalidated manometry protocol, and inability to perform several maneuvers demanding cooperation from patients. However, to date, the use of ARM to predict the therapeutic response in children is not fully understood or described and has been identified as a research priority in the field of Neurogastroenterology and Motility [1,2]. We hypothesize that ARM helps in predicting therapeutic response in children with functional constipation and present our experience elucidating the same.

## 2. Methods

### 2.1. Study Design

This retrospective chart review study was conducted at two tertiary centers. Approval for the study was obtained from the Institutional Review Boards at both centers, and appropriate data agreements were established. Data were collected retrospectively from electronic health records (EHR) of pediatric patients who underwent Anorectal Manometry (ARM) between 1 January 2018 and 1 July 2022. Both centers had the equipment manufactured by Laborie/MMS system.

### 2.2. Participants

Pediatric patients who met the Rome IV criteria for functional constipation and underwent ARM during the study period were included. Patients with a known cause of constipation, such as Hirschsprung’s disease, spinal abnormalities, or colorectal malformations, were excluded.

### 2.3. Data Collection

Demographic and clinical variables, including study indication, pharmacotherapy (e.g., laxative regimen), interventions (e.g., anal Botox), and duration and response to interventions, were collected from electronic health records (EHR). ARM variables recorded included resting anal pressure (mmHg), presence of recto anal inhibitory response (RAIR) and the percentage of relaxation at each incremental balloon inflation volume from 10 cc to 60 cc, squeeze effort (mmHg and percentage increase from baseline), first rectal sensation with balloon inflation (volume in cc), and presence of dyssynergic defecation on bear-down maneuver (BDM). BDM data were included for patients older than 8 years of age and when the study was performed without sedation. Given the retrospective nature of study, the standardization of the protocol was not conducted. However, both participating centers follow the maneuvers described in the ANMS consensus document [1].

### 2.4. Response to Therapy

We recorded therapeutic interventions (osmotic and stimulant laxatives) and response to therapy was deemed as successful when there was an increase in frequency of bowel movements to 3 or more per week and/or a decrease/improvement in frequency of fecal incontinence (FI) to <1 episode per week. We also evaluated the potential association (or predictive value) of ARM on eventual need for surgery at last follow-up.

### 2.5. Statistical Analysis

Demographic factors and ARM parameters were compared with response to therapy and eventual need for surgery. Statistical analysis was performed using the Statistical Package for the Social Sciences (SPSS V 28.0). Continuous variables were expressed as medians (range) and were compared using non-parametric tests, while proportions were compared using Chi-square or Fisher’s exact tests. Multivariate logistic regression models were utilized to determine factors potentially associated with outcomes.

## 3. Results

A total of 327 patients with functional constipation undergoing an ARM at two tertiary motility centers (Saint Louis University and Yale University) were included; median age was 8.2 years (range 1–20 years), with 25.7% of those including young children (<6 y of age), 49.5% school-age children (6–12 y of age) and 24.8% % were adolescents (>12 y of age); 175/327 (54%) were female and 152/327 (46%) were male. Of those 327 patients, 140 received an increased dose of an osmotic laxative as the first treatment after the ARM, 156 a daily dose of stimulant laxatives, 19 secretagogues, 6 received irritant softeners, and 6 prucalopride. The overall response to all medications was 133/327 or 40.7%. A total of 232 patients received a daily dose of a stimulant laxative after the ARM, 156 as the first and the rest as the second intervention. The overall response to stimulant laxatives was 112/232 or 48%.

### 3.1. Association Between Demographics and Response to Therapy

We found no association between gender and response to all therapies and stimulant laxatives (*p* = 0.681 and *p* = 0.496, respectively), but we did find an association between older age and response to all therapies (median age of 9 in responders vs. 8 in non-responders, *p* = 0.018) and response to stimulant laxatives (median age of 8.8 years in responders vs. 7.8 years in non-responders, *p* = 0.046).

### 3.2. Association Between ARM Parameters and Response to Therapy

*Anal resting pressure.* The median IAS resting pressure for all groups was 68 mmHg (range 12–161 mmHg). We found that responders to any therapy had a significantly lower anal resting pressure (*p* = 0.01), and we found a tendency towards an association between lower anal resting pressure and response to stimulant laxatives (*p* = 0.07). See Table 1.

RAIR presence. RAIR was absent or equivocal in 38 (11.6%) patients. We found no association between response to therapy and the presence or absence of RAIR. See Table 1.

IAS percentage of relaxation. We found no association between the median percentage of relaxation at all volumes tested (10–60 cc) and response to any therapy. See Table 1.

Squeeze maneuver. The median squeeze increase in pressure was 154 mmHg (range 50–315 mmHg), and the median percentage increase was 94% (range 10–444%). We found an association between a higher percentage of squeeze pressure increase from baseline and lack of response to any therapy (*p* = 0.026) and a tendency towards an association between the overall increase in pressure in mmHg from baseline and lack of response to any therapy (*p* = 0.09). We found no association between squeeze maneuver parameters and response to stimulant laxatives. See Table 1.

First rectal sensation. Median first rectal sensation was 7 mmHg (range 5–240 mmHg). We found an association between response to therapy and higher first rectal sensation. See Table 1.

Bear-down maneuver. The bear-down maneuver was evaluated in 83 patients, and it was found to be abnormal in 45/83 or 54% of patients. We found an association between abnormal bear-down maneuver and lack of response to all therapies; those with normal BDM responded and failed equally (50%) to all therapies, but those with abnormal BDM failed all therapies at a higher rate (78%), *p* = 0.008. We also observed a higher failure response to stimulant laxatives (68%) in those with abnormal BDM compared to those with normal BDM (61%), *p* = 0.036. See Table 1.

### 3.3. Eventual Need for Surgery

A total of 47 (14%) patients failed all medical therapy and eventually required surgery (38 underwent an ACE procedure, 4 had a distal colon resection, and 5 had a diverting ostomy). Age and gender were not associated with the eventual need for surgery (*p* = 0.372 and *p* = 0.496, respectively). We found that no patient with abnormal BDM (45) eventually underwent surgery compared to 4 of 38 with normal BDM (*p* = 0.04). We found that 11/38 (29%) with abnormal RAIR eventually required surgery compared with 36/289 (12%) with normal RAIR (*p* = 0.006). No other ARM parameter was associated with the eventual need for surgery.

Multivariate analysis. Logistic regression demonstrated no demographic factor nor ARM parameter were associated with response to therapy (all therapies or stimulant laxatives). See Table 2. Excluding those that received sedation or using logistic regression to evaluate the confounding effect of sedation does not change the results except only for the percentage of increase in pressure from baseline in squeeze maneuver for all therapy (*p* = 0.002 unadjusted vs. *p* = 0.058 when adjusted for sedation).

## 4. Discussion

Anorectal manometry (ARM) is the most performed motility test in pediatric patients. The techniques and equipment utilized in ARM have undergone significant advances, facilitating performance and drastically improving the fidelity of the data acquired [3]. However, there is still significant variability in the performance of the procedure within different institutions [4], and little is known about its utility in predicting therapeutic outcomes, particularly in children with functional constipation [5]. In this retrospective study, we report our experience from two centers to explore the utility of ARM in children with functional constipation to predict and guide therapeutic strategies.

We found that responders to any therapy had a lower anal resting pressure than non-responders, suggesting less outflow resistance may lead to better outcomes, although both values (responders and non-responders) were within normal range, so the significance of this observation is unclear. In a recent study evaluating the effect of non-standardized medical therapy on ARM parameters, El-Shabrawi et al. reported a non-statistically significant increase in anal resting pressure despite a decrease in rectal sensation parameters after medical therapy [6], while Keshtgar et al. reported hypertrophic changes and thickening of the internal anal sphincter (IAS) and rectal wall with longstanding constipation and fecal retention [7], potentially explaining some of the changes in resting pressure in chronic constipation and suggesting it could be a finding unrelated to therapy response and rather to chronic constipation. Given that Staller et al. have also reported that resting anal pressure is the single most important factor on ARM in predicting healthcare utilization in adults [8], our finding merits further exploration. We also found an association between a higher percentage of squeeze pressure increase from baseline (but not for the delta in mmHg) and lack of response to all therapies, suggesting non-responders could generate higher squeeze pressures, a finding potentially associated with withholding behavior, which could also explain the lack of response on those patients to conventional therapy that does not address potential behavioral component. El-Shabrawi et al. reported non-statistically significant lower squeeze pressures after therapy but no mention of the predictive value of the ARM parameters in therapeutic response [6]. Our findings also suggest that responders have delayed rectal sensation, which could explain why they responded to therapy that may improve rectal sensation parameters, as has been reported by El-Shabrawi [6].

Perhaps the most important contribution of our findings is the association between abnormal BDM and a lack of response to various laxative therapies, suggesting the role of anorectal manometry (ARM) in age-appropriate patients (<8–9 years) who exhibit normal development and cooperation during ARM to detect dyssynergic defecation that requires specific therapy (other than conventional stool softeners and stimulant laxatives) including biofeedback and potentially anal botulinum toxin injections on those failing all other interventions [9,10,11,12]. We also found that no patient with abnormal BDM required surgery, and those with absent or abnormal RAIR were more likely to require surgery (29%) compared to those with normal RAIR (12%), suggesting ARM can potentially predict the eventual need for surgery in specific populations.

Our study does have inherent limitations. It was retrospective, with an important proportion of patients being excluded due to incomplete electronic health records (EHR), and medical treatments were not standardized. Many results acquired from the ARM study are also age-dependent, and it is possible that the results of some of the maneuvers are partially inaccurate, given patients’ ability to understand and follow the instructions. The sequential effects of laxative medications were not consistently documented, and potential errors may have arisen from EHR documentation by providers and recall bias from patients. Designing a prospective study to evaluate the sequential impact of laxatives and to obtain accurate ARM data to correlate clinical and manometric information represents the next desirable step.

## 5. Conclusions

This is the first study examining the clinical utility of ARM in predicting therapeutic response in pediatric patients with FC. We found those with lower anal resting pressure, lower squeeze effort, and delayed rectal sensation respond better to medical therapy. ARM identified those with abnormal bear-down maneuvers and predicted a lack of response to conventional therapy without the need for eventual surgical interventions. In addition to diagnosing HD, ARM could be an instrumental tool in identifying patients with dyssynergic defecation for early intervention with targeted therapy in age-appropriate patients.

## Figures and Tables

**Table 1:** Univariate analysis evaluating association between ARM parameters and response to therapy.

	Overall Response					
	All Medications			Stimulant Laxatives		
	Yes	No	*p*	Yes	No	*p*
Resting Pressure						
Median (mmHg)	61.5	70	0.01	57.5	64	0.070
IAS Median Relaxation (%)						
10 cc	20.5	26	0.693	16	20	0.778
20 cc	34	40	0.552	36	38	0.810
30 cc	37	44	0.452	37	42	0.824
40 cc	47.5	49	0.506	40.5	49	0.094
50 cc	53.5	58	0.559	49	52.5	0.983
60 cc	52	59	0.258	52.5	50	0.799
Squeeze Maneuver (Median)						
Increase from baseline (mmHg)	137	163	0.091	133	149	0.125
Increase from baseline (%)	67	101	0.026	69	96	0.379
1st rectal sensation median volume (cc)	5	10	0.002	6	10	0.002
RAIR	N (%)	N (%)				
Normal n (%)	115/289 (40)	174/289 (60)	0.371	95/199 (48)	104/199 (52)	0.668
Abnormal n (%)	18/38 (47)	20/38 (53)		17/33 (52)	16/33 (48)	
Bear-down maneuver	N (%)	N (%)		N (%)	N (%)	
Normal n (%)	19/38 (50)	19/38 (50)	0.008	14/23 (61)	9/23 (39)	0.036
Abnormal n (%)	10/45 (22)	35/45 (78)		10/31 (32)	21/31 (68)	

**Table 2:** Multivariate analysis to evaluate joint effect of demographics and ARM findings on response to therapy.

	Response to Therapy							
	All Therapies				Stimulant Laxatives			
			95% CI				95% CI	
	*p*	OR	Lower	Upper	*p*	OR	Lower	Upper
Age (years)	0.072	1.440	0.967	2.143	0.109	1.495	0.914	2.445
Female gender	0.536	0.513	0.062	4.252	0.693	1.745	0.110	27.644
Abnormal BDM	0.631	0.565	0.055	5.795	0.337	0.237	0.012	4.482
Anal resting pressure (mmHg)	0.691	0.988	0.930	1.049	0.133	0.927	0.840	1.023
Squeeze increase (mmHg)	0.672	0.993	0.961	1.026	0.900	1.003	0.963	1.044
Rectal sensation (cc)	0.746	0.952	0.705	1.285	0.566	0.865	0.527	1.420

CI: confidence interval.

## Data Availability

The original contributions presented in the study are included in the article, further inquiries can be directed to the corresponding author.
